# Effect of Blood Sampling Time After Colostrum Intake on the Concentration of Metabolites Indicative of the Passive Immunity Transfer in Newborn Dairy Calves

**DOI:** 10.3390/ani14213133

**Published:** 2024-10-31

**Authors:** Mohammad Hassan Mortazavi, Nathalia Brito Rocha, Marília Ribeiro de Paula, Evangelina Miqueo, Marcia Saladini Vieira Salles, Paulo Henrique Mazza Rodrigues, Carla Maris Machado Bittar

**Affiliations:** 1 Department Animal Science, University of Tehran, Karaj 1417935840, Iran; mohamadmortazavi@ut.ac.ir; 2 Department of Animal Sciences, College of Agriculture “Luiz de Queiroz”, Av. Pádua Dias, 11, Piracicaba 13418-900, SP, Brazil; n.britorocha@yahoo.com.br (N.B.R.); marilia.rp@hotmail.com (M.R.d.P.); evangelina.miqueo@gmail.com (E.M.); 3 Sao Paulo Agency for Agribusiness Technology (APTA), Ribeirao Preto 14030-670, SP, Brazil; marcia.salles@sp.gov.br; 4 Department Animal Nutrition and Production, Faculty of Veterinary Medicine and Animal Science, Av. Duque de Caxias Norte, 225, Pirassununga 13635-900, SP, Brazil; pmazza@usp.br

**Keywords:** blood parameters, health monitoring, immunoglobulin, refractometer, total serum protein

## Abstract

This study aimed to evaluate the impact of time after birth on the concentrations of metabolites relevant to monitoring passive immune transfer in calves. Additionally, we aimed to identify the best measurement devices based on accuracy, speed, cost-effectiveness, and availability on farms. Calves received 6 L of high-quality colostrum within 6 h. Blood samples were taken before (0 h) and 1, 2, 4, 6, 12, 24, 48, 72, 96, and 120 h after colostrum intake. The total serum protein (TSP) concentration increased during the first hours after colostrum intake, reaching stability between 24 and 48 h. The γ-glutamyl transferase and alkaline phosphatase concentrations increased after 4 h, followed by a significant decrease, and stabilized after 24 h. The albumin concentration decreased from 0 to 12 h then increased until 48 h and stabilized. Hematocrit showed higher values one hour after intake and remained constant after 24 h. Total serum protein stabilized at 24–48 h, which was the optimal window for passive transfer assessment. Digital or optical refractometers are recommended for TSP measurement, showing a strong correlation with enzyme methods, though initial calibration is necessary due to brand variations. These devices offer faster results compared to enzyme methods and direct IgG analysis.

## Simple Summary

Newborn calves are agammaglobulinemic, meaning that they are born without the protective antibodies that help protect against infections. As a result, the transfer of passive immunity through colostrum becomes crucial for the health and survival of these calves, significantly impacting their morbidity and mortality rates, and their growth. This study investigated the best sampling time and compared methods for determining the total serum protein and the dynamics of other metabolites, which enable us to monitor potential failures in passive immunity transfer. The best time to measure total serum protein was between 24 and 48 h after birth, and the most cost-effective and fastest measuring device was a digital or optical refractometer.

## 1. Introduction

Due to the synepitheliochorial placental structure, bovines show no maternal antibody flow from mother to fetus during pregnancy, resulting in an agammaglobulemic newborn [1]. Therefore, passive immunity transfer (PIT) is an important issue for the calf, affecting morbidity and mortality rates, and growth rates [2,3]. In a comparative analysis of calves with total TSP levels < 5.1 g/dL and those with >6.2 g/dL, the incidence rates of diarrhea, pneumonia, and pre-weaning mortality were found to be 1.5, 1.4, and 4.29 times higher in the lower TSP group, respectively [4]. The volume of colostrum intake may even influence productive life in adulthood [5]. Colostrum is composed not only of immunoglobulins, but also of other proteins, fats, lactose, cytokines, growth factors, and substantial numbers of maternal leukocytes [6].

Adequate colostrum feeding protocols are based on three important factors: the feeding time, quality, and volume fed. Combined, these factors should result in a consumed minimum dose of 150 to 200 g IgG of colostrum or colostrum replacement products in the first 24 h [7]. Otherwise, the calf may present a failure of the passive transfer of immunity (FPTI) [8]. Calves are defined as having FPTI if the calf serum IgG concentration is lower than 10 g/L [9,10,11] or the serum protein level is less than 5.2 g/dL [9,12,13] in a dichotomy approach. However, the most recent recommendations regarding colostrum feeding management suggest that 40% of calves should present IgG levels higher than 25 g/L, 30% between 18 and 24.9 g/L, 20% between 10 and 17.9 g/L, and less than 10% lower than 10 g/L [14]. The efficiency of Ig absorption is initially high in newborn calves, but gradually declines over time. This decline continues until approximately 24 h of life, coinciding with the maturation of intestinal cells and the development of the intracellular digestive apparatus, a process called intestinal closure [15].

The average total cost per dairy calf affected by FPTI is estimated to be EUR 60 (ranging from EUR 10 to EUR 109), with specific scenarios indicating costs as low as EUR 52 in the best case and as high as EUR 285 in the worst case; this cost estimation suggests an approximately 50% increase for beef calves, highlighting the significant economic effects of FPTI on calf rearing systems [16]. For analysis, passive immune transfer monitoring requires blood samples from the newborn calf after birth. The determination of circulating colostral immunoglobulin in the serum of animals may be performed by laboratory analysis (direct methods) using different methods such as radial immune diffusion (RID), turbidimetric immunoassays, the enzyme-linked immunosorbent assay (ELISA), the transmitted and attenuated total reflectance infrared (ATR) spectroscopic method, the split trehalase IgG assay (STIGA), electrophoresis, capillary electrophoresis (CE), and proteomics [17]. However, these methods are costly and unpractical in commercial herds. Alternative indirect methods can estimate circulating Ig by reading the TSP in the calf’s serum using a protein [17,18,19,20] or a brix refractometer [17,18,21]. Several studies have shown that the serum Ig concentration is highly correlated with the TSP of calves less than 7 days old, measured in a laboratory [18,21,22] or brix refractometer [18,21,23].

Serum protein fractions are associated with colostrum intake and age; thus, age must be considered when evaluating TSP as a marker for FPTI [24]. Sampling calves to diagnose FPTI when this correlation is no longer strong may lead to false negatives and the incorrect perception that the colostrum protocol is efficient in a particular farm. Other proteins, such as γ-glutamyl transferase (GGT) and alkaline phosphatase (AP), also have a positive correlation with immunoglobulins, allowing these enzyme concentrations to be used to evaluate the passive immune status of calves [22,25].

On dairy farms, particularly in the context of calf management, the rapid assessment of Ig levels in the blood is crucial for evaluating the passive immune status of calves. Given the high correlation between indirect and direct measurement methods, indirect techniques are frequently employed due to their cost-effectiveness and efficiency. The objective of this study was to systematically assess the influence of the time after birth on the concentrations of metabolites relevant to monitoring passive immune transfer in calves. Additionally, this study aimed to identify the most practical metabolites and measurement devices in terms of accuracy, speed, cost-effectiveness, and availability, and establish the optimal time for sampling to facilitate the effective monitoring of the FPTI.

## 2. Materials and Methods

### 2.1. Animal Ethics

The Animal Ethics Committee of the College of Agriculture, University of São Paulo, approved all animal procedures (Protocol 2014-18).

### 2.2. Animals

Forty-seven male (n = 22) and female (n = 25) newborn calves from the herd of the University of Sao Paulo, College of Agriculture ‘Luiz de Queiroz’, Department of Animal Science (USP; n = 36, birth weight = 32.8 ± 1.00 kg, Holstein × Gir), and from the herd of the Sao Paulo Agency for Agribusiness (APTA; n = 11, birth weight = 24.0 ± 1.53 kg, Jersey × Gir), were used.

### 2.3. Feeding and Management

Calving was monitored, and the calves were separated from their multiparous dams just after birth to avoid suckling. They were weighed on a mechanical scale (ICS-300, Coimma Ltda., Sao Paulo, SP, Brazil) and housed in individual hutches. The navel was treated with iodine 7% at birth and then at least twice a day until complete dehydration and loss of the cord. Fresh cows were milked just after parturition, and the colostrum quality was monitored by a digital brix refractometer (Hanna Instruments, HI 96811, Woonsocket, Rhode Island, RI, USA) and a colostrometer (Nasco, Fort Atkinson, WI, USA). If colostrum was not adequate in volume or quality (<22% Brix or <50 mg Ig/mL), high-quality colostrum from a colostrum bank was thawed in a water bath (<55 °C). The calves received 6 L of high-quality colostrum (>22% Brix, [26]), split into two feedings within 6 h after birth. In cases of intake refusal (4 male calves), the calves were tubed to ensure the total amount of colostrum intake. At approximately 12 h after birth, calves were fed 4 L of low-quality colostrum (<30 mg Ig/mL or <15% Brix). From the second day of life, calves were fed 4 L/d of whole milk.

### 2.4. Blood Parameters

Blood samples were collected from calves at 0 h (before), 1 h, 2 h, 4 h, 6 h, 12 h, 24 h, 48 h, 72 h, 96 h, and 120 h after colostrum intake. Jugular venipuncture was performed using a clot-activating tube to determine TSP, albumin, GGT, and alkaline phosphatase (ALP). The samples were centrifuged (Universal 320R, Hettich, Tuttlinger, Germany) at 2000× *g* for 20 min at 4 °C to obtain serum, and stored at −26 °C in plastic microtubes until subsequent analysis. Hematocrit was determined using an aliquot of blood containing anticoagulant, filling up 3/4 of the capillary tube without heparin (Classcyto^®^, Sao Paulo, Brazil), using a microcentrifuge hematocrit SPIN model 1000 (Microspin, Model Spin 1000, Sao Paulo, Brazil) at 12,000× *g* for 10 min.

The determination of TSP using a handheld protein refractometer (TSP_ref_-Instrutemp, Model ITREF 200, Sao Paulo, Brazil) was performed at all sampling times. Specific commercial enzymatic kits (Labtest Diagnóstica S.A., Minas Gerais, Brazil) were used to analyze the total serum protein (TSP_enz_), albumin, GGT, and ALP, using an Automatic System for Biochemistry (CELM, SBA 200, Sao Paulo, Brazil).

### 2.5. Statistical Analysis

Data were statistically analyzed using the SAS 9.4 (SAS Institute Inc., Cary, NC, USA). Before the main analysis, the data were analyzed for the presence of outliers, as well as the normality of residuals. An individual observation was considered an outlier when the standard deviation in relation to the mean was higher than +3 or lower than −3. The normality of residuals was accessed through the Shapiro–Wilk test at Univariate Procedure of SAS (PROC UNIVARIATE). Analyses of variance were performed according to the PROC MIXED model, with the collection times used as repeated measures. The best covariance structure was identified from 15 different covariance structures by comparing the Akaike Information Criteria Corrected (AICC) statistic [27]. The model included, as fixed effects, sex (male and female), sampling time and the interaction between these factors; as a covariate, this model included the birth body weight, time to colostrum feeding and colostrum mass ingested up to 12 h. The block effect (Farms USP and APTA) was included in the model as a random effect, and consequently controlled for breed factor. All the selected metabolites and hematocrit were analyzed as repeated measures over time: Y_ijk_ = μ + D_i_ + b_j_ + e_ij_ + I_k_ + (D_i_)_Ik_ + e_ijk_, where μ = overall mean; D_i_ = fixed effect of sex; b_j_ = random effect of block; e_ij_ = residual error (A); I_k_ = fixed effect of sampling time; (D_i_)I_k_ = effect of sex × effect of sampling time interaction; and e_ijk_ = residual error (B). Mean comparisons among times were carried out according to the adjusted Tukey test, with an adopted significance level of 5%. The correlations between the TSP analyzed by the enzymatic procedure or estimated by the refractometer and sampling time were explored through PROC CORR of SAS.

## 3. Results

**Figure 1:**
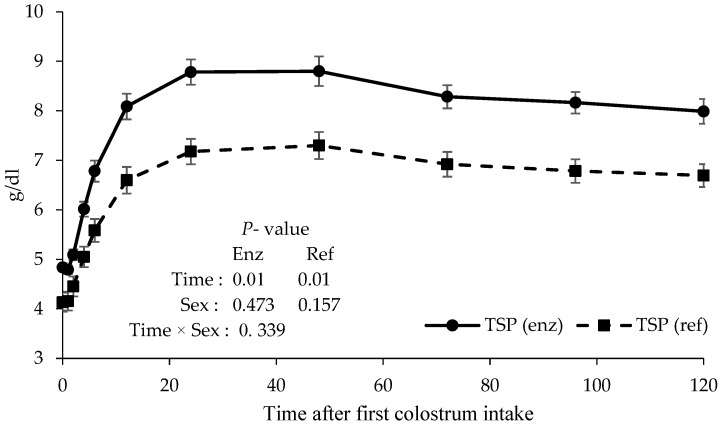
Total serum protein, determined by the refractometer (Ref) or enzymatic method (Enz), of newborn calves according to the time after colostrum intake.

The total serum protein concentration, measured by either TSP_ref_ or TSP_enz_, increased during the first hours of life (Figure 1). The average concentrations of TSP_ref_ and TSP_enz_ at 24 h after colostrum intake were 7.18 ± 0.25 g/dL and 8.78 ± 0.26 g/dL, respectively. The maximum concentration and subsequent stabilization of TSP occurred between 24 and 48 h following colostrum intake (TSP_enz_ reaching its peak at 24 h and TSP_ref_ at 48 h), and no significant difference was observed between them. Both concentrations differed significantly from those measured at earlier sampling intervals (*p* < 0.05). However, there was a significant decrease between 72 and 96 h compared to 48 h, but these were not significantly lower than at 24 h. The animal sex did not affect TSP_ref_ and TSP_enz_ according to the time after colostrum intake.

The regression analysis of TSP showed quadratic behavior for both the refractometer and enzymatic methods of protein determination (Table 1). Compared to conventional enzyme methods or direct IgG analysis, the utilization of optical or digital refractometers costs approximately USD 1 and USD 12 per sample, respectively.

**Figure 2:**
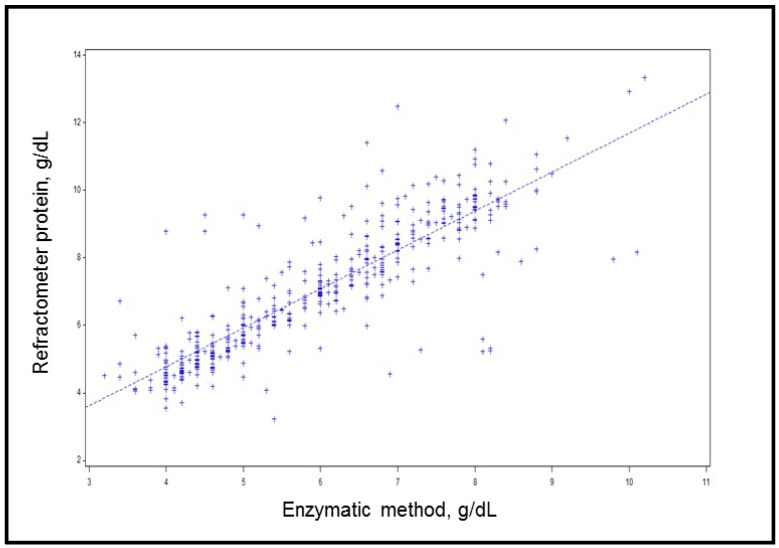
Correlation between methods of total protein determination in the serum of dairy calves.

The values of TSP_enz_ were higher than those of TSP_ref_ (Figure 1; *p* < 0.05) but presented a similar time behavior and a high positive correlation between the refractometer and enzymatic methods (r = 0.75; *p* < 0.05) (Table 1; Figure 2).

**Figure 3:**
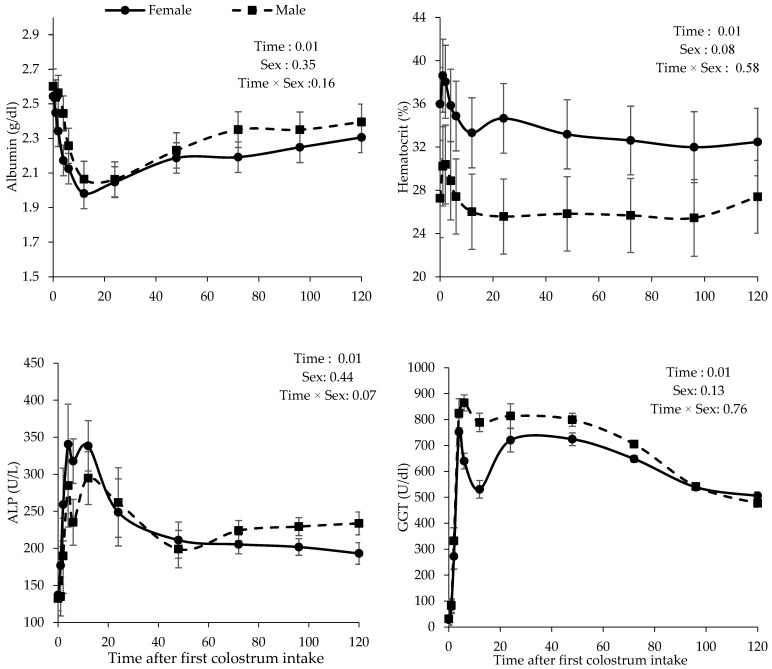
Albumin, hematocrit GGT, and ALP concentrations in the blood of newborn calves according to the time after colostrum intake.

Up to 24 h after the first colostrum intake, the albumin concentration decreased and then increased (Figure 3). At 24 h, the albumin concentration tended to be significantly lower compared to 6 h (*p* < 0.10), and it was not significantly different from 12 h. At other times, a significant difference was observed. There was no significant difference among 48, 72, 96, and 120 h. Animal sex did not affect the albumin concentration according to the time after colostrum intake.

Hematocrit was affected by the time after colostrum intake (*p* < 0.01), with higher values observed at one hour after colostrum intake (Figure 3), but there was a significant difference between one hour and all other sampling times. There was no significant difference among 24, 48, 72, 96, and 120 h. There was a difference between animal sex in the hematocrit parameter, which tended to be significant. Females had higher hematocrit concentrations than males according to the time after colostrum intake.

As shown in Figure 3, the ALP concentration increased from 0 h to 6 h after colostrum intake (*p* < 0.01). Additionally, a significant difference was observed at 24 h compared to 0 h, as well as at 48 h in comparison to both 0 h and 12 h. No significant difference was observed among 24, 48, 72, 96, and 120 h. Animal sex did not affect ALP according to the time after colostrum intake.

The GGT concentration was significantly different over time after colostrum intake. The concentration of GGT reached its peak 4 h after colostrum intake, then decreased until 12 h, and then increased and stabilized after 24 h, but no significant difference was observed among 4, 6, 12, 24, and 48 h. The sampling times of 4, 6, 24, and 48 h differed significantly from 0, 1, 2, and 120 h. The gamma-glutamyl transferase concentration at 120 h significantly decreased compared to 72 h. Animal sex did not affect GGT according to the time after colostrum intake.

## 4. Discussion

Immunoglobulins constitute a significant portion of the circulating proteins in the serum of newborn calves, while the levels of non-immune proteins remain relatively consistent across individuals. Consequently, assays that estimate the total protein concentrations in serum or plasma have been widely employed to diagnose FPTI [21,25,28]. Several cut-point values for diagnosing FPTI have been proposed, with TSP concentrations ranging from 5.0 to 5.5 g/dL [12,18,28]. It was concluded that to reduce the incidence of false negative cases specifically, namely the number of calves with FPTI that are not identified by the test, a cut-off point of 5.5 g/dL for total protein refractometry is more appropriate than the previously suggested threshold of 5.2 g/dL. This recommendation is based on a review of studies utilizing total protein refractometers [29]. The minimum TSP concentrations should be higher than 6.2 g/dL in 40 % of the calves, between 5.8 and 6.1 in 30 % of the calves, between 5.1 and 5.7 in 20 % of the calves, and lower than 5.1 in only 10 % of the calves [14]. In the current study, the highest TSP concentration was observed between 24 and 48 h. This period coincides with the 24 and 48 h age of the animals, which is characterized by the complete closure of the absorption of colostrum protein [2,15] and represents the time required for the systemic absorption and circulation of colostral antibodies until they appear in the animal serum [30]. Other studies also show an increased TSP concentration from the first day after birth [31] and the maintenance of high values during the first week of life [32]. It was observed that there was no significant difference in the TSP parameter until the third day of life [33].

The optical or digital protein refractometer quantifies the refractive index changes induced by the constituents of serum samples [34]. A light beam is directed through the serum sample positioned within a prism in this measurement process. The proteins present in the serum interact with the light, causing refraction. Notably, the degree of light refraction is directly proportional to the protein concentration within the sample [35]. Considering this aspect, the quadratic behavior observed for the refractometer and enzymatic evaluation of TSP suggests that the maximum time after colostrum intake for the accurate evaluation of passive immunity transfer is 24–48 h (Table 1). Therefore, it is important to consider the age at which passive immunity transfer is evaluated. Because TSP_ref_ (g/dL) is an indirect determination, being the value calculated from the refractive index (nD) and a conversion factor specific to each refractometer model, TSP_enz_ was higher than TSP_ref_. However, this difference does not invalidate the use of the refractometer due to the similar time behavior and high positive correlation (r = 0.75; *p* < 0.05) between the two methods (Table 1; Figure 2). A high correlation exists between the TSP concentrations obtained from the protein refractometer readings and the IgG levels analyzed through RID, thereby corroborating the use of this tool for the evaluation of FPTI [18,36].

A positive correlation exists between the serum albumin concentration and colostrum intake in newborn calves. Ensuring that calves receive sufficient high-quality colostrum shortly after birth is vital for boosting serum albumin levels, supporting their immune function, and promoting overall health and development [37]. The average albumin concentration was within the reference values for calves during the first 5 days of age [38,39]. In the current study, the highest average concentration (2.56 g/dL ± 0.07) was observed before colostrum intake (0 h) and the lowest (2.02 g/dL ± 0.07) at 12 h after the first meal of colostrum. In one [24] study, it was observed that the highest albumin concentration at birth was followed by a decrease and a subsequent increase in albumin concentrations. Considering the TSP variation, it is possible to suppose that the differences in the albumin concentration are due to colostrum immunoglobulin’s intestinal absorption. Albumin is responsible for 75% of the osmotic activity, and its increase with age is physiological and important for maintaining metabolic balance [24]. Thus, the decreasing values observed within the first 12 h of colostrum intake do not express the absolute albumin concentration and may be the result of the osmotic pressure of protein fractions in plasma. This occurs because of plasma volume expansion resulting from colostrum intake and its metabolism during the first hours of life [40].

The quantification of TSP concentrations is significantly constrained by the influence of dehydration on the accuracy of the results. Consequently, this assay must be conducted on clinically healthy calves to ensure reliable and valid results [21]. However, in the present study, animals had hematocrit within the expected values for calves at this stage of life [38]. The hematocrit concentration reached a peak in the first hour after colostrum intake. This could have resulted from colostrum intake and its metabolism during the first hour of life [41]. After the maximum concentration, there was a decrease, probably because of the osmotic pressure effect of protein fractions in plasma [42].

Gamma-glutamyl transferase is an enzyme that plays a crucial role in various biological processes in calves, particularly in liver function and glutathione metabolism. GGT is involved in transferring gamma-glutamyl groups from glutathione and other peptides, which is significant for detoxification processes and maintaining cellular redox status [43]. Upon ingesting colostrum, the calf absorbs the mother-originated GGT, leading to its detection in the serum. The serum GGT activity in colostrum-fed calves is significantly elevated, ranging from 60 to 160 times higher than that in healthy adult cattle [44]. Among the proteins absorbed from the colostrum by the calf intestine, GGT presents a close correlation with the Ig concentration [45,46]. The concentration of GGT increased from the lowest average (29.84 ± 11.33 U/L) at birth to its maximum at about 6 h after colostrum intake (752.21 ± 61.10 U/L), with small variations related to the second colostrum meal (Figure 3). The concentrations subsequently decreased to 491.76 ± 43.96 U/L at 120 h after the colostrum intake. This finding is consistent with the results reported by others [47,48], indicating a correlation between colostrum intake and the observed changes in enzyme levels over time. The results obtained for the GGT and ALP concentrations are related to colostrum intake, since the concentration peak coincides with the moment showing the greatest absorption of macromolecules in newborn calves. The relationship between the ALP levels and colostrum intake in newborn calves indicates growth, nutritional status, and metabolic health. Ensuring that calves receive adequate amounts of high-quality colostrum shortly after birth is vital for their development and overall health, which can also be reflected in their ALP levels [49]. These proteins, together with TSP, could improve the monitoring of FPTI [25,50].

## 5. Conclusions

Metabolites related to colostrum intake have an important effect depending on the colostrum feeding time. Total serum protein reaches stability at 24–48 h, being the best time window for passive transfer evaluation. Besides total serum protein, other proteins may be used to monitor passive immune transfer and improve the colostrum feeding protocols. Based on the results and the high correlation between the enzymatic method and the refractometer estimates, measuring TSP in farms using a digital or optical refractometer is an important tool for understanding passive transfer. Furthermore, using these devices accelerates the generation of results compared to conventional enzyme methods or direct IgG analysis and saves money.

## Figures and Tables

**Table 1:** Regression equations of total serum protein (TSP), measured by the enzymatic method or by the protein refractometer, with time after colostrum intake by dairy calves.

Regression Equations	R^2^	*p*-Value
TSP_ref_ = 5.304999 + 0.01931 T	0.2876	0.01
TSP_ref_ = 4.79507 + 0.08244 T − 0.00058577 T^2^	0.5176	0.01
TSP_enz_ = 6.13537 + 0.02468 T	0.2486	0.01
TSP_enz_ = 5.53535 + 0.10027 T − 0.00070451 T^2^	0.4233	0.01
TSP_enz_ = 0.1662 + 1.1517 TSP_ref_	0.7518	0.01

## Data Availability

The original contributions presented in the study may be available upon request to the corresponding author.

## References

[1] Peter A.T. (2013). Bovine placenta: A review on morphology, components, and defects from terminology and clinical perspectives. Theriogenology.

[2] Godden S. (2008). Colostrum management for dairy calves. Vet. Clin. N. Am. Food Anim. Pract..

[3] Lora I., Gottardo F., Contiero B., Ava B.D., Bonfanti L., Stefani A., Barberio A. (2018). Association between passive immunity and health status of dairy calves under 30 days of age. Prev. Vet. Med..

[4] Crannell P., Abuelo A. (2023). Comparison of calf morbidity, mortality, and future performance across categories of passive immunity: A retrospective cohort study in a dairy herd. J. Dairy Sci..

[5] Urie N., Lombard J., Shivley C., Kopral C., Adams A., Earleywine T., Olson J., Garry F. (2018). Preweaned heifer management on US dairy operations: Part V. Factors associated with morbidity and mortality in preweaned dairy heifer calves. J. Dairy Sci..

[6] McGrath B.A., Fox P.F., McSweeney P.L., Kelly A.L. (2016). Composition and properties of bovine colostrum: A review. Dairy Sci. Technol..

[7] Godden S.M., Lombard J.E., Woolums A.R. (2019). Colostrum management for dairy calves. Vet. Clin. Food Anim. Pract..

[8] Kehoe S., Jayarao B., Heinrichs A. (2007). A survey of bovine colostrum composition and colostrum management practices on Pennsylvania dairy farms. J. Dairy Sci..

[9] Chigerwe M., Hagey J.V., Aly S.S. (2015). Determination of neonatal serum immunoglobulin G concentrations associated with mortality during the first 4 months of life in dairy heifer calves. J. Dairy Res..

[10] Quigley Iii J., Kost C., Wolfe T. (2002). Absorption of protein and IgG in calves fed a colostrum supplement or replacer. J. Dairy Sci..

[11] Weaver D.M., Tyler J.W., VanMetre D.C., Hostetler D.E., Barrington G.M. (2000). Passive transfer of colostral immunoglobulins in calves. J. Vet. Intern. Med..

[12] Hernandez D., Nydam D., Godden S., Bristol L., Kryzer A., Ranum J., Schaefer D. (2016). Brix refractometry in serum as a measure of failure of passive transfer compared to measured immunoglobulin G and total protein by refractometry in serum from dairy calves. Vet. J..

[13] Tyler J.W., Hancock D.D., Parish S.M., Rea D.E., Besser T.E., Sanders S.G., Wilson L.K. (1996). Evaluation of 3 assays for failure of passive transfer in calves. J. Vet. Intern. Med..

[14] Lombard J., Urie N., Garry F., Godden S., Quigley J., Earleywine T., McGuirk S., Moore D., Branan M., Chamorro M. (2020). Consensus recommendations on calf-and herd-level passive immunity in dairy calves in the United States. J. Dairy Sci..

[15] Quigley Iii J., Drewry J. (1998). Nutrient and immunity transfer from cow to calf pre-and postcalving. J. Dairy Sci..

[16] Raboisson D., Trillat P., Cahuzac C. (2016). Failure of passive immune transfer in calves: A meta-analysis on the consequences and assessment of the economic impact. PLoS ONE.

[17] de Souza R.S., Dos Santos L.B.C., Melo I.O., Cerqueira D.M., Dumas J.V., Leme F.d.O.P., Moreira T.F., Meneses R.M., de Carvalho A.U., Facury-Filho E.J. (2021). Current diagnostic methods for assessing transfer of passive immunity in calves and possible improvements: A literature review. Animals.

[18] Deelen S., Ollivett T., Haines D., Leslie K. (2014). Evaluation of a Brix refractometer to estimate serum immunoglobulin G concentration in neonatal dairy calves. J. Dairy Sci..

[19] Kaneko J.J., Harvey J.W., Bruss M.L. (2008). Clinical Biochemistry of Domestic Animals.

[20] Quigley J.D., Lago A., Chapman C., Erickson P., Polo J. (2013). Evaluation of the Brix refractometer to estimate immunoglobulin G concentration in bovine colostrum. J. Dairy Sci..

[21] Cuttance E., Regnerus C., Laven R. (2019). A review of diagnostic tests for diagnosing failure of transfer of passive immunity in dairy calves in New Zealand. New Zealand Vet. J..

[22] Šlosárková S., Fleischer P., Pěnkava O., Skřivánek M. (2014). The assessment of colostral immunity in dairy calves based on serum biochemical indicators and their relationships. Acta Vet. Brno.

[23] Morrill K., Polo J., Lago A., Campbell J., Quigley J., Tyler H. (2013). Estimate of serum immunoglobulin G concentration using refractometry with or without caprylic acid fractionation. J. Dairy Sci..

[24] Tóthová C., Nagy O., Kováč G., Nagyová V. (2016). Changes in the concentrations of serum proteins in calves during the first month of life. J. Appl. Anim. Res..

[25] Lima P.P.A., Alcindo J.F., Fioruci J.C.R., Costa L.R., de Oliveira P.L., Bosculo M.R.M., Grassi T.L.M., Ponsano E.H.G., Ferreira C.Y.M.R., de Almeida B.F.M. (2024). Passive immunity transfer in bovine calves: Analysis methods and their correlations with maternal and colostral parameters. Comp. Clin. Pathol..

[26] Bielmann V., Gillan J., Perkins N., Skidmore A., Godden S., Leslie K. (2010). An evaluation of Brix refractometry instruments for measurement of colostrum quality in dairy cattle. J. Dairy Sci..

[27] Wang Z., Goonewardene L.A. (2004). The use of MIXED models in the analysis of animal experiments with repeated measures data. Can. J. Anim. Sci..

[28] Cuttance E., Mason W., Denholm K., Laven R. (2017). Comparison of diagnostic tests for determining the prevalence of failure of passive transfer in New Zealand dairy calves. New Zealand Vet. J..

[29] Buczinski S., Gicquel E., Fecteau G., Takwoingi Y., Chigerwe M., Vandeweerd J. (2018). Systematic review and meta-analysis of diagnostic accuracy of serum refractometry and brix refractometry for the diagnosis of inadequate transfer of passive immunity in calves. J. Vet. Intern. Med..

[30] Villarroel A., Miller T., Johnson E., Noyes K., Ward J. (2013). Factors affecting serum total protein and immunoglobulin G concentration in replacement dairy calves. Adv. Dairy Res..

[31] Mann S., Curone G., Chandler T., Sipka A., Cha J., Bhawal R., Zhang S. (2020). Heat treatment of bovine colostrum: II. Effects on calf serum immunoglobulin, insulin, and IGF-I concentrations, and the serum proteome. J. Dairy Sci..

[32] Wilm J., Costa J.H., Neave H.W., Weary D.M., von Keyserlingk M.A. (2018). Serum total protein and immunoglobulin G concentrations in neonatal dairy calves over the first 10 days of age. J. Dairy Sci..

[33] Silper B., Coelho S., Madeira M., Ruas J., Lana A., Reis R., Saturnino H. (2012). Colostrum quality evaluation and passive immunity transfer in crossbred Holstein Zebu cattle. Arq. Bras. de Med. Veter. E Zootec..

[34] Tothova C., Nagy O., Kovac G. (2016). Serum proteins and their diagnostic utility in veterinary medicine: A review. Veter. Med..

[35] Quigley J. (2006). Calf Note# 39—Using a Refractometer. https://www.calfnotes.com/pdffiles/CN039.pdf.

[36] Elsohaby I., McClure J., Keefe G. (2015). Evaluation of digital and optical refractometers for assessing failure of transfer of passive immunity in dairy calves. J. Vet. Intern. Med..

[37] Hadorn U., Hammon H., Bruckmaier R.M., Blum J.W. (1997). Delaying colostrum intake by one day has important effects on metabolic traits and on gastrointestinal and metabolic hormones in neonatal calves. J. Nutr..

[38] Knowles T., Edwards J., Bazeley K., Brown S., Butterworth A., Warriss P. (2000). Changes in the blood biochemical and haematological profile of neonatal calves with age. Vet. Rec..

[39] Rauprich A., Hammon H., Blum J. (2000). Influence of feeding different amounts of first colostrum on metabolic, endocrine, and health status and on growth performance in neonatal calves. J. Anim. Sci..

[40] Elsohaby I., Mweu M.M., Mahmmod Y.S., McClure J.T., Keefe G.P. (2019). Diagnostic performance of direct and indirect methods for assessing failure of transfer of passive immunity in dairy calves using latent class analysis. Prev. Vet. Med..

[41] Hopkins B., Quigley J. (1997). Effects of method of colostrum feeding and colostrum supplementation on concentrations of immunoglobulin G in the serum of neonatal calves. J. Dairy Sci..

[42] Flores R., Souza C., Ocarino N., Gheller V., Lopes M., Palhares M., Serakides R. (2006). Hypertonic and isotonic saline solutions in dehydration therapy in neonate calves: Comparison of clinical profile and serum and urinary concentrations of electrolytes. Comp. Clin. Pathol..

[43] Hoffmann W.E., Solter P.F. (2008). Diagnostic enzymology of domestic animals. Clin. Biochem. Domest. Anim..

[44] Güngör Ö., Bastan A., Erbıl M. (2004). The usefulness of the γ-glutamyltransferase activity and total proteinemia in serum for detection of the failure of immune passive transfer in neonatal calves. Rev. de Med. Vet..

[45] Gibson I. Failure of passive transfer. Proceedings of the Society of Dairy Cattle Veterinarians of the New Zealand Veterinary Association Annual Conference.

[46] Pekcan M., Fıdancı U.R., Yuceer B., Ozbeyaz C. (2013). Estimation of passive immunity in newborn calves with routine clinical chemistry measurements. Ank. Üniversitesi Vet. Fakültesi Derg..

[47] Kurz M., Willett L. (1991). Carbohydrate, enzyme, and hematology dynamics in newborn calves. J. Dairy Sci..

[48] Ribeiro De Paula M., Brito Rocha N., Miqueo E., Moura Silva F.L., Gavanski Coelho M., Machado Bittar C.M. (2019). Passive immune transfer, health, pre-weaning performance, and metabolism of dairy calves fed a colostrum supplement associated with medium-quality maternal colostrum. Rev. Bras. de Zootec..

[49] Rocha T.G., Nociti R.P., Sampaio A.A., Fagliari J.J. (2012). Passive immunity transfer and serum constituents of crossbred calves. Pesqui. Veter. Bras..

[50] Osaka I., Matsui Y., Terada F. (2014). Effect of the mass of immunoglobulin (Ig) G intake and age at first colostrum feeding on serum IgG concentration in Holstein calves. J. Dairy Sci..

